# Circ_0001786 facilitates gefitinib resistance and malignant progression in non-small cell lung cancer via miR-34b-5p/SRSF1

**DOI:** 10.1186/s13019-024-02651-9

**Published:** 2024-04-05

**Authors:** Kaobin Ouyang, Dan Xie, Haojie Liao, Ying He, Hailin Xiong

**Affiliations:** Department of Medical Oncology, Huizhou Municipal Central Hospital of Guangdong Province, NO.41 North Eling Road, Huizhou, 516000 Guangdong Province China

**Keywords:** NSCLC, circ_0001786, miR-34b-5p, SRSF1, Gefitinib

## Abstract

**Background:**

Non-small cell lung cancer (NSCLC) is a widespread cancer and gefitinib is a primary therapy for NSCLC patients. Nevertheless, the underlying mechanisms for the progression of acquired drug resistance have not been clarified. The aim of this study was to investigate the role of circular RNA (circ_0001786) in gefitinib-resistant NSCLC.

**Methods:**

Firstly, the expression of circ_0001786, miR-34b-5p and SRSF1 were assayed using qRT-PCR. Subsequently, CCK-8 test was utilized to measure the semi-inhibitory concentration (IC50) of cellular gefitinib. Apoptosis was identified by flow cytometry. At last, dual luciferase assay was applied to prove the binding association between miR-34b-5p, circ_0001786 or SRSF1.

**Results:**

Our research disclosed that circ_0001786 was heightened in gefitinib-resistant NSCLC cells and tissues. Knockdown of circ_0001786 restrained IC50 values of gefitinib, attenuated the clonogenic ability and facilitated apoptosis in HCC827-GR and PC9-GR. In addition, circ_0001786 was a molecular sponge for miR-34b-5p. Silencing miR-34b-5p rescued the inhibitory impact of circ_0001786 knockdown on IC50 and cell cloning ability. Moreover, miR-34b-5p directly targeted SRSF1. Importantly, circ_0001786 enhanced gefitinib tolerance and malignant development in NSCLC through miR-34b-5p/SRSF1 pathway.

**Conclusion:**

This research revealed a novel mechanism by which circ_0001786 enhanced NSCLC resistance to gefitinib by sponging miR-34b-5p and upregulating SRSF1. circ_0001786 was a potential target for improving the treatment of gefitinib-resistant NSCLC patients.

## Introduction

Lung cancer is a prevalent neoplasm worldwide, with the highest tumor incidence and death rate [1]. Non-small cell lung cancer (NSCLC) is the major form of lung cancer, taking account for approximately 85% [2]. Regardless of the remarkable advance in the therapy of lung cancer at this stage in point of means, equipment and technology, the prognosis of patients is still poor. Epidermal growth factor receptor (EGFR) mutations are found in approximately 20 to 40% of these NSCLC patients and promote cancer progression [3]. The advent of the molecularly targeted drug gefitinib has led to a breakthrough in the remedy of NSCLC. Gefitinib is a first-generation EGFR-tyrosine kinase inhibitors (EGFR-TKIs) and is the first TKI drugs permitted by the FDA for the treatment of patients with advanced NSCLC [4]. It inhibited cellular angiogenesis and cell growth and proliferation by competitively binding to the ligand-binding site located outside the cell with Adenosine triphosphate, blocking the phosphorylation process of intracellular tyrosine kinases, and further attenuating the activation of the EGFR pathway. Owing to its outstanding efficacy and minimal harmful side effects, it has become the primary therapy option for patients with EGFR-sensitive mutant NSCLC. Nevertheless, with the prolongation of medication, the drug resistance of gefitinib gradually increased, many patients develop resistance within one year after starting treatment, leading to treatment failure. Therefore, elucidating the mechanism of gefitinib resistance in NSCLC and finding ways to reverse resistance have become urgent issues.

There is evidence that sustained stimulation of NSCLC cells by gefitinib causes alterations in a variety of cancer-associated circular RNAs (circRNAs), leading to reduced drug effectiveness [5]. circRNAs are generated by reverse splicing of precursor mRNA transcripts [6]. As a novel endogenous non-coding RNA, circRNAs are broad, conserved, and stable [7]. CircRNAs are closely linked to the growth, invasion and apoptosis of many tumor cells, containing lung cancer, and expected to be a prognostic marker for cancer therapy [8]. It has been shown that a few circRNAs play important roles in NSCLC by serving as tumor inhibitors or enhancers. For example, circATXN7 is heightened in NSCLC, circATXN7 silencing hampers growth and migration of NSCLC cells [9]. In addition, circRNAs are taken part in the formation of tumor drug resistance. For instance, circPTK2 alleviates cellular cisplatin resistance via targeting the miR-942/TRIM16 pathway in NSCLC [10]. Circ_0011298 improves paclitaxel resistance in NSCLC via sponging miR-486-3p and raising CRABP2 [11]. Circ_0001786 is a freshly identified circRNA, which is aberrantly shown in lung cancer and may be associated to the development of lung cancer, whereas its role in lung cancer is still unclear.

Numerous researches have manifested that circRNA is associated with the advancement of certain diseases by regarding as a sponge for microRNA (miRNA). For example, circNRIP1 performs as a miRNA-149-5p sponge and promotes the development of gastric cancer through the AKT1/mTOR axis [12]. MiRNAs are a single-stranded non-coding RNAs that play a part in gene regulation through mRNA degradation or translation [13]. Dysregulation of miRNA expression is nearly related to human cancer development. miR-196b-5p is upregulated in NSCLC, and its mediated inhibition of TSPAN12 and GATA6 accelerates the development in NSCLC [14]. miRNAs are also participated in the advancement of tumor medicine tolerance. miR-1914-3p [15], miR-138-5p [16], miR-524-5p [17] and other miRNAs have been proved to adjust drug tolerance in NSCLC. miR-34b-5p is aberrantly shown in various diseases. It was discovered that miR-34b-5p promotes apoptosis through regulation of granulin precursors in acute lung injury mouse model exacerbated by LPS [18]. The lncRNA TUG1 attenuates sepsis-induced infectious lung injury through combining with miR-34b-5p [19]. Nevertheless, the regulatory mechanism of miR-34b-5p in NSCLC has not been elucidated.

The objective of this research was to clarify the potential mechanism of circ_0001786 in gefitinib resistance NSCLC. Our outcomes disclosed that circ_0001786 was enhanced in gefitinib-resistant NSCLC. Silencing of circ_0001786 facilitated the susceptibility of NSCLC to gefitinib. Additionally, we researched the connections between circ_0001786, miR-34b-5p and SRSF1 in NSCLC gefitinib resistance and found that circ_0001786 regulated NSCLC gefitinib resistance through miR-34b-5p/SRSF1 axis. This study may provide fresh therapy approaches for NSCLC patients with gefitinib resistance.

## Materials and methods

### Clinical NSCLC samples

40 clinical specimens of NSCLC and normal paracancerous tissues were gathered. All patients were diagnosed with NSCLC by histopathology. The tissues were divided based on the patients’ response to gefitinib: gefitinib-sensitive group and gefitinib-resistant group. Liquid nitrogen was used to freeze the tissues immediately, which were subsequently placed at -80 °C refrigerator. All subjects voluntarily signed the informed consent form.

### Cell line culture and transfection

NSCLC cell lines (PC9, HCC827), human bronchial epithelial cells 16HBE were bought from the Chinese Academy of Sciences (Shanghai, China). Gefitinib-resistant cells (PC9-GR, HCC827-GR) were created by exposing PC9 and HCC827 to progressively raising dosages of gefitinib. Each concentration gradient was incubated in medium for at least 2 weeks until the cells maintained good viability. All cells were cultured in RPMI-1640 with 10% FBS (Gibco, USA) at 37 °C containing 5% CO_2_.

Specific circ_0001786 (si-circ_0001786), si-NC, miR-34b-5p inhibitor, miR-34b-5p mimic, miR-NC, pcDNA SRSF1 and pcDNA-NC were produced by GenePharma commercially available. Cells were transfected with plasmids or oligonucleotides by Lipofectamine 2000 reagent and transfection capability was verified using qRT-PCR after 48 h.

### Cell viability assay (CCK-8)

Transfected cells (4 × 10^3^) were seeded into 96-well plates. 24 h later, the cells were exposed to diverse dosages of gefitinib and further incubated for 48 h. Then, 10 µL of CCK-8 solution was mixed and cultured for 1 h at 37 °C. The optical density of each well was examined by absorbance at 450 nm using an enzyme marker.

### Cell clone experiments

Transfected cells (2 × 10^2^) were inoculated on 6-well plates and cultivated for 2 weeks. After that, the cells were stained with 4% paraformaldehyde and 0.5% crystalline violet for 20 min, respectively. Images were obtained under a light microscope and visible colonies were counted.

### Apoptosis assay

Transfected cells (5 × 10^4^) were seeded into 6-well plates and cultivated for 72 h. Apoptosis assays were performed using an apoptosis kit (Invitrogen, USA). Briefly, 500 µL of binding buffer (Thermo Scientific, USA) was used to resuspend the collected cells. This buffer contained 5 µL of membrane-linked Annexin-V PE and AAD (Sigma-Aldrich, USA). Cells were cultivated for 20 min. The quantity of apoptotic cells was measured by flow cytometry.

### qRT-PCR assay

We used Trizol reagent to extract total RNA. Subsequently, cDNA was synthesized by Superscrpt II kit (Invitrogen, USA). Then, SYBR Premix Ex Taq II /ABI 7500 StepOnePlus system (California, USA) was performed to measure circRNA and mRNA levels. Primer sequences were as below (5’→3’):

circ_0001786: F: ACTCCTGACACTGAAGATCCAG;

R: CCAATGATGCAGCCACTTGTCA.

miR-34b-5p: F: GGGTAGGCAGTGTCATTAGC;

R: AACAACCACACACAACACAC.

SRSF1: F: ATGTCGGGAGGTGGTGAT;

R: TCTGCTTCTCCTTGGGGT.

GAPDH: F: AAGGT-GAAGGTCGGAGTC;

R: AAGATGGTGAT-GGGGATTTC.

U6: F: CTCGCTTCGGCAGCACA;

R: AACGCTTCACGAATTTGCGT.

### Dual-luciferase reporter assay

According to the bioinformatics website, the potential site miR-34b-5p for circ_0001786 and the potential site SRSF1 for miR-34b-5p were forecasted. The combined sequences were cloned, then they were inserted into pmirGLO to create the wild-type reporter vector circ_0001786-WT and SRSF1-WT, and mutant reporter vectors circ_0001786-MUT and SRSF1-MUT. The circ_0001786-WT, circ_0001786-MUT or miR-NC mimic, miR-34b-5p mimic were transfected into cells. Similarly, SRSF1-WT, SRSF1-MUT or miR-NC mimic, miR-34b-5p mimic were transfected into cells. Afterwards, the relative luciferase activity in the cells was measured by a commercial dual luciferase reporter kit (Promega, USA).

### Western blot (WB) assay

We utilized RIPA lysis buffer to isolate total protein from cells or tissues. 30 µg of total protein was taken for SDS-PAGE at a steady voltage of 100 V. The target protein bands were transferred to PVDF membranes at a steady flow of 250 mA. Then, the membranes were closed with 5% milk and incubated with primary antibody (1:1000) overnight at 4 °C, followed by incubation with the corresponding secondary antibody (1:1000) for 1 h at room temperature. Finally, the ECL system was used to visualize protein bands and quantified them using Image J.

### Statistical analysis

The information was processed by GraphPad Prism 7.0. To compare differences, Student’s t-test was used. Correlation analysis was applied using Spearman analysis. *p*-value was used to determine statistical significance. The symbols were used: *, *p* < 0.05; **, *p* < 0.01; ***, *p* < 0.001; ns indicated not significant.

## Results

### circ_0001786 is enhanced in gefitinib-resistant NSCLC

**Fig. 1:**
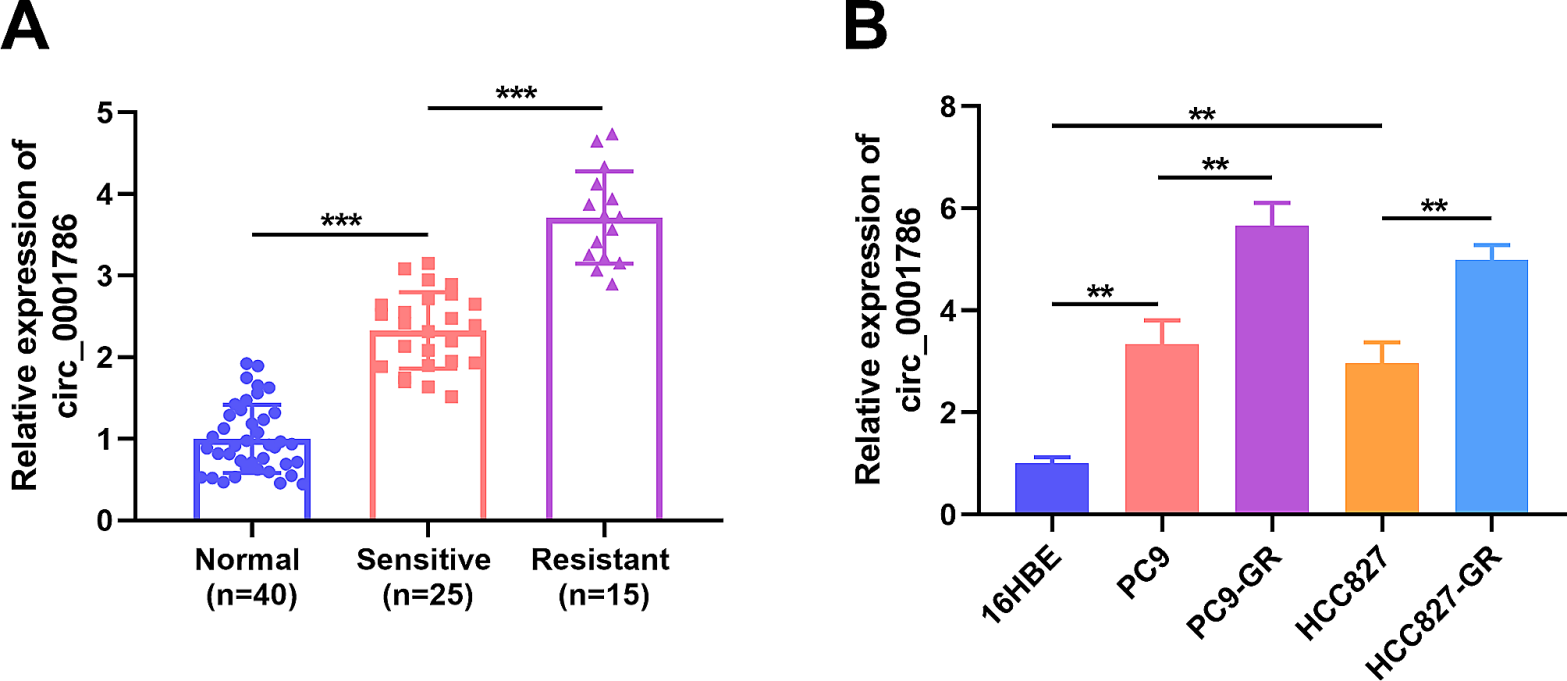
circ_0001786 expression was associated with Gefitinib-resistance in NSCLC. (**A**) circ_0001786 expression was measured by qRT-PCR in 25 primary gefitinib-sensitive NSCLC tissues, 15 gefitinib-resistant NSCLC tissues (recurrent) and 40 matched adjacent lung tissues. (**B**) circ_0001786 expression in 16HBE, PC9, HCC827, PC9-GR and HCC827-GR cells was detected by qRT-PCR. **P < 0.01, ***P < 0.001

circ_0001786 was assessed by qRT-PCR to investigate its effect on gefitinib resistance in NSCLC. We disclosed that circ_0001786 were enlarged in NSCLC tissues. In addition, the level of circ_0011298 was magnified in gefitinib-resistant tissues compared to gefitinib-sensitive tissues (Fig. 1A). Moreover, circ_0001786 was dramatically heightened in PC9 and HCC827 contrasted to 16HBE, while the circ_0001786 expression was obviously strengthened in gefitinib-resistant cells relative to PC9 and HCC827 (Fig. 1B). The results suggested that circ_0001786 participated in the progression of gefitinib tolerance in NSCLC. Next, to evaluate the pathological significance of circ_0001786 in the tissue expression of NSCLC, we analyzed the clinicopathological characteristics of the patients. The outcomes showed that circ_0001786 was clearly correlated with tumor stage, tumor size, and lymph node metastasis, whereas there was not significantly related to age, sex, smoking, or histological type (Table 1).

**Table 1:** Correlations between circ_0001786 and clinical characteristics of 40 NSCLC patients

Characteristics	Total Number	circ_0001786 expression		
		Low(*n* = 20)	High(*n* = 20)	*P* value
Age	40			> 0.9999
≤ 60		10	11	
> 60		10	9	
Sex	40			0.7475
Male		11	13	
Female		9	7	
Smoking	40			> 0.9999
Yes		13	14	
No		7	6	
Histological Type	40			> 0.9999
Squamous Cell Carcinoma		6	7	
Adenocarcinomas		14	13	
Tumor stage	40			0.0104*
I + II		14	5	
III + IV		6	15	
Tumor size	40			0.0012**
≤ 3		16	5	
> 3		4	15	
Lymph node metastasis	40			0.0248*
No		13	5	
Yes		7	15	

### Knockdown of circ_0001786 improves the sensitivity of gefitinib

**Fig. 2:**
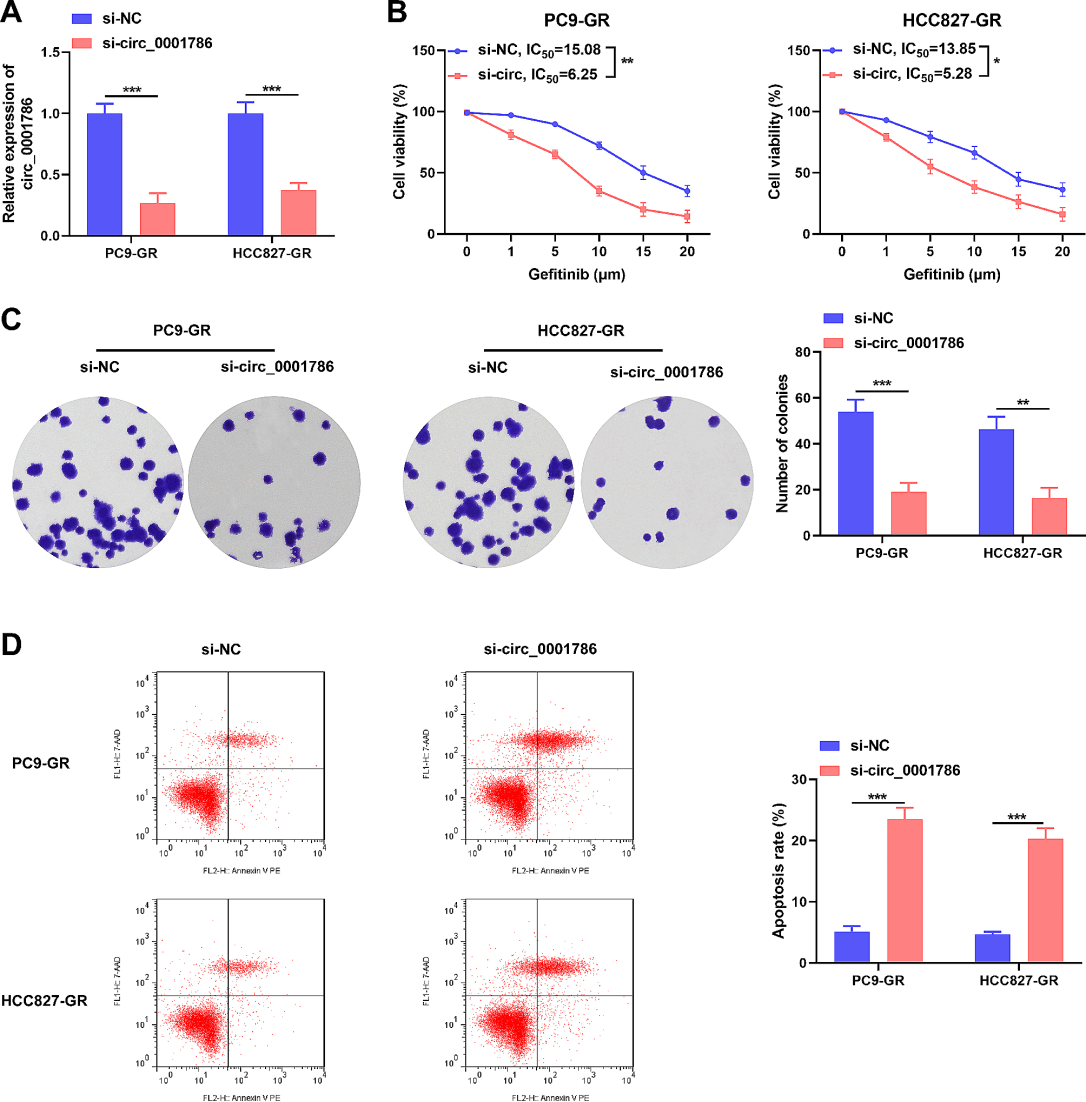
circ_0001786 knockdown suppressed cell colony formation and promoted gefitinib sensitivity in Gefitinit-resistant NSCLC cell line. (**A**) circ_0001786 knockdown efficiency in PC9-GR and HCC827-GR cells was checked by qRT-PCR. (**B**) Cell viability of transfected cells was detected by CCK8 assay after treatment with the indicated concentration of gefitinib. (**C**) Cell colony formation ability was detected by colony formation assay after circ_0001786 knockdown in PC9-GR and HCC827-GR cells. (**D**) Cell apoptosis was measured by flow cytometry after circ_0001786 knockdown PC9-GR and HCC827-GR cells. *P < 0.05, **P < 0.01, ***P ＜0.001

To evaluate the function of circ_0001786, functional assays were carried out in gefitinib-resistant cells. The results disclosed that circ_0001786 expression was depressed in cells transfected with si-circ_0011298 contrasted to si-NC, implying that the transfection was successful (Fig. 2A). Notably, knockdown of circ_0001786 restrained the IC50 values of gefitinib (Fig. 2B), indicating that silencing of circ_0001786 sensitized NSCLC cells to gefitinib. Meanwhile, circ_0001786 inhibition hampered the colony-forming ability of cells, revealing that circ_0001786 silencing inhibited cell proliferation ability (Fig. 2C). In addition, circ_0001786 knockdown considerably induced the apoptotic capacity of gefitinib-resistant cells (Fig. 2D). Together, the outcomes disclosed that circ_0001786 regulated the gefitinib sensitivity in NSCLC cell lines.

### circ_0001786 targets miR-34b-5p

**Fig. 3:**
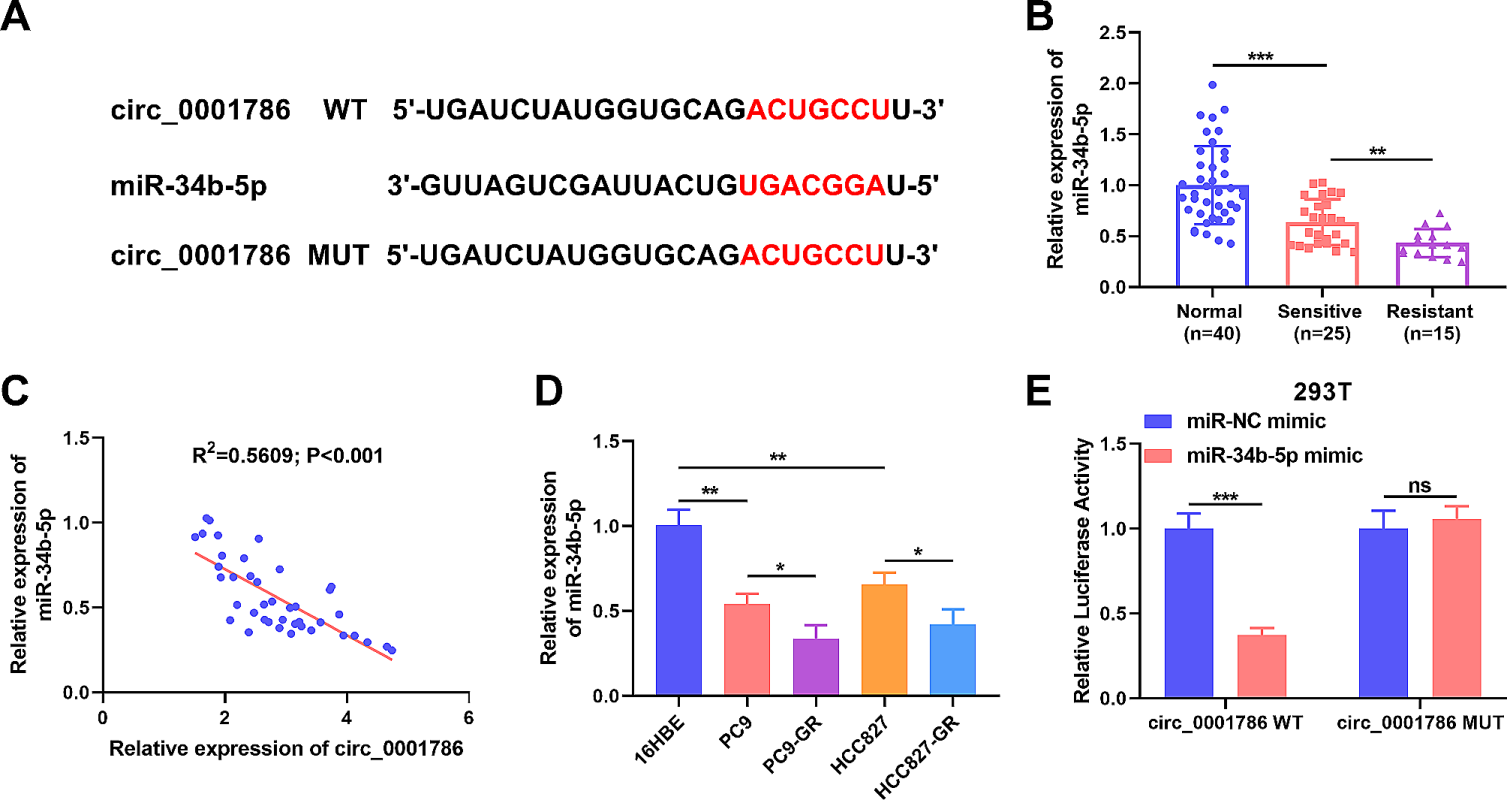
circ_0001786 can bind with miR-34b-5p. (**A**) The putative binding sequence between circ_0001786 and miR-34b-5p. (**B**) The level of miR-34b-5p in NSCLC tissues was detected by qRT-PCR. (**C**) Correlation between miR-34b-5p and circ_0001786 level in 40 NSCLC tissues using Spearman analysis. (**D**) The miR-34b-5p expression in 16HBE, PC9, HCC827, PC9-GR and HCC827-GR cells was detected by qRT-PCR. (**E**) Dual-luciferase assay was introduced in 293 T cells to confirm the binding relationship between circ_0001786 and miR-34b-5p. *P < 0.05, **P < 0.01, ***P < 0.001

To clarify the value of circ_0001786, we used bioinformatics analysis of public databases to predict the downstream targets of circ_0001786. A binding site between circ_0001786 and miR-34b-5p was disclosed (Fig. 3A). Next, miR-34b-5p levels were analyzed by qRT-PCR in normal and gefitinib-resistant/sensitive NSCLC tissue. The findings displayed that miR-34b-5p was declined in gefitinib-sensitive NSCLC tissue samples contrasted with normal tissues, and miR-34b-5p was further diminished in gefitinib-resistant tissues (Fig. 3B). Spearman analysis discovered an inverse association between miR-34b-5p and circ_0001786 levels in gefitinib-resistant tissues (R^2^ = 0.5609, *p* < 0.001) (Fig. 3C). Then, miR-34b-5p level in NSCLC cells was assessed by qRT-PCR. The outcomes found that miR-34b-5p was clearly depressed in PC9 and HCC827 compared to 16HBE, while miR-34b-5p was dramatically prevented in gefitinib-resistant cells contrasted to PC9 and HCC827 (Fig. 3D). Subsequently, luciferase assay revealed that miR-34b-5p mimic degraded the luciferase intensity in circ_0001786 WT, whereas no such change was detected in circ_0001786 MUT group (Fig. 3E). The findings manifested that circ_0001786 bound specifically to miR-34b-5p.

### Knockdown of circ_0001786 regulates gefitinib sensitivity via miR-34b-5p

**Fig. 4:**
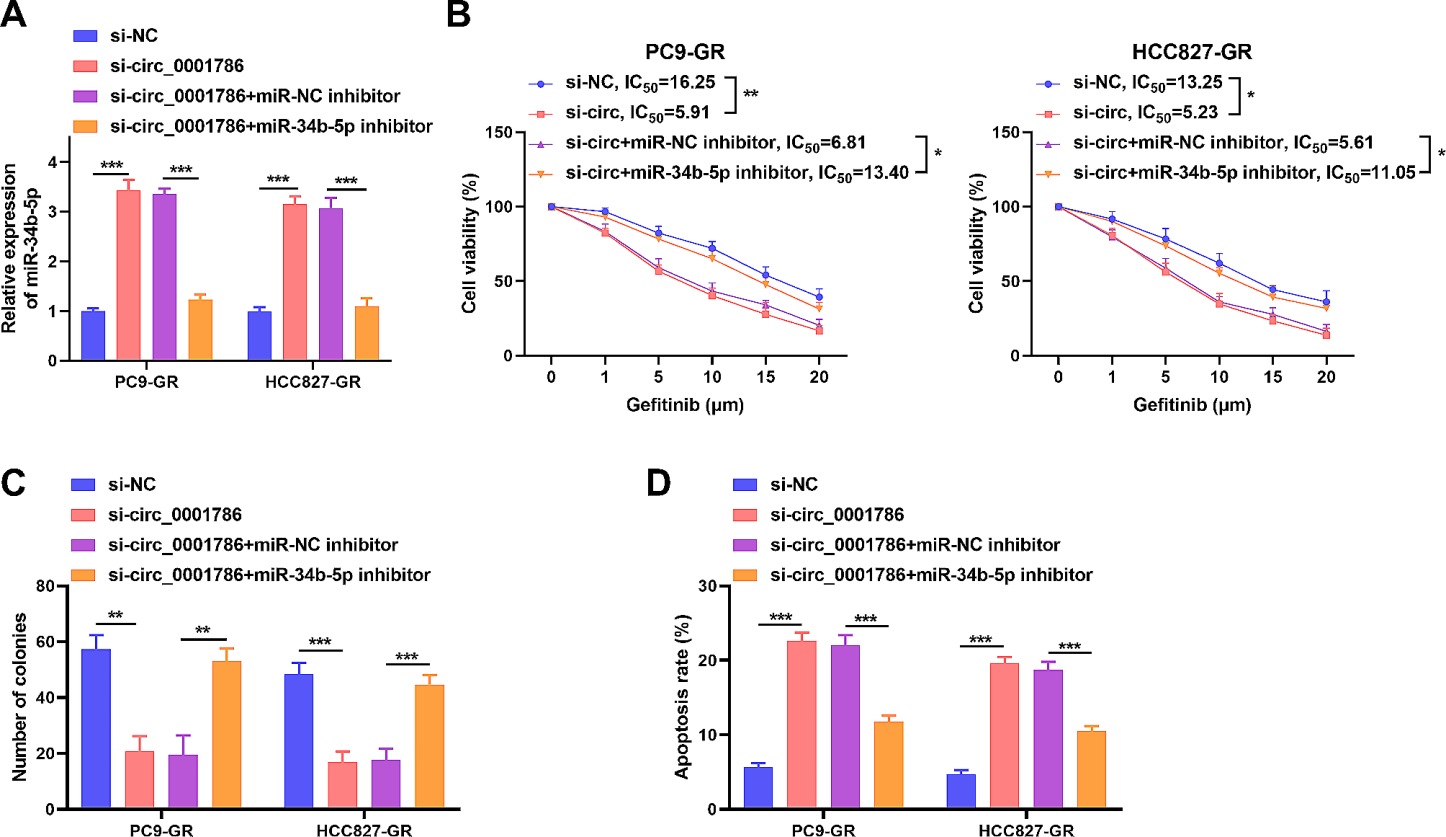
circ_0001786 silencing regulated gefitinib-resistant NSCLC cell colony formation ability, apoptosis and gefitinib sensitivity by targeting miR-34b-5p. PC9-GR and HCC827-GR cells were transfected with si-NC, si-circ (si-circ_0001786), si-circ + inhibitor NC or si-circ + miR-34b-5p inhibitor. (**A**) miR-34b-5p expression after different treatments. (**B-D**) Cell viability, colony formation ability and apoptosis were detected by CCK8 (**B**), colony formation assay (**C**) and flow cytometry (**D**) after different treatments. *P < 0.05, **P < 0.01, ***P < 0.001

To evaluate the specific worth of circ_0001786 and miR-34b-5p in regulating gefitinib resistance, a rescue assay was performed. We cotransfected PC9-GR and HCC827-GR cells with si-circ_0001786 and miR-34b-5p inhibitor. Results disclosed that miR-34b-5p in cells was raised after circ_0001786 silencing, while miR-34b-5p inhibitor returned the change, indicating that the transfection was well (Fig. 4A). Functional assays revealed that circ_0001786 knockdown restrained the IC50 value and cell cloning ability in gefitinib-resistant cells, which offset by miR-34b-5p inhibitor (Fig. 4B, C). Meanwhile, silencing of circ_0001786 enhanced the apoptotic capacity of gefitinib in PC9-GR and HCC827-GR, whereas the process was abolished by miR-34b-5p inhibitor (Fig. 4D). These data illustrated that circ_0001786 adjusted gefitinib sensitivity via miR-34b-5p.

### miR-34b-5p targets SRSF1

**Fig. 5:**
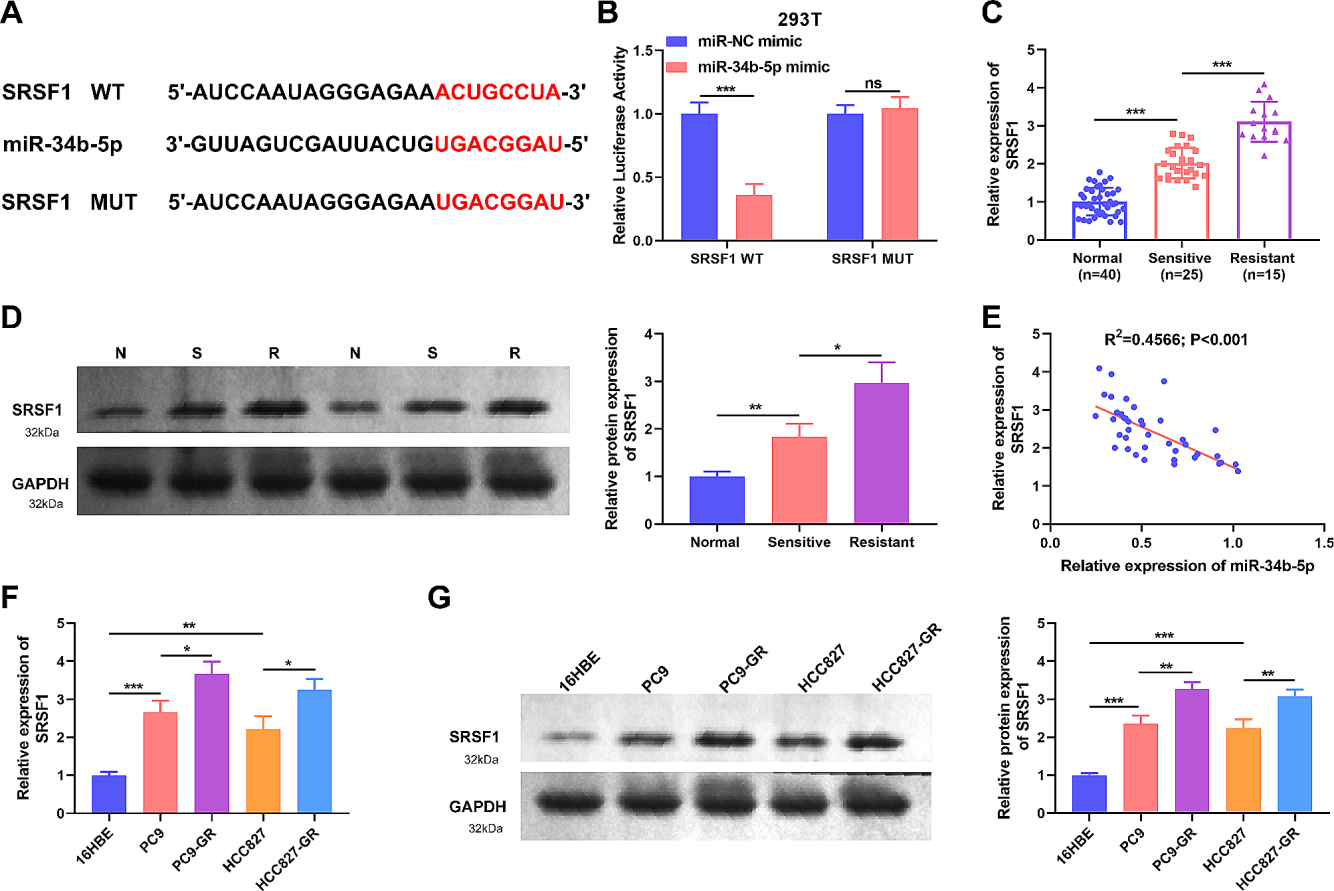
miR-34b-5p directly targeted the 3’UTR of SRSF1. (**A**) The putative binding sequence between miR-34b-5p and 3’UTR of SRSF1. (**B**) Dual-luciferase assay was used in 293 T cells to confirm the binding relationship between miR-34b-5p and SRSF1. (**C-D**) The level of SRSF1 in NSCLC tissues was detected by qRT-PCR(**C**) and western blot (**D**). (**E**) Correlation between miR-34b-5p and SRSF1 level in 40 NSCLC tissues using Spearman analysis. (**F-G**) The SRSF1 RNA expression in 16HBE, PC9, HCC827, PC9-GR and HCC827-GR cells was detected by qRT-PCR (**F**) and western blot (**G**). *P < 0.05, **P < 0.01, ***P < 0.001

To research the mechanism of miR-34b-5p on gefitinib resistance in NSCLC, the target of miR-34b-5p was explored. We found that SRSF1 had a binding site to miR-34b-5p (Fig. 5A). Dual luciferase assay discovered that transfection of miR-34b-5p mimic noticeably inhibited the luciferase activity of SRSF1-WT, nevertheless, the luciferase activity of SRSF1-MUT was not obviously affected, demonstrating the connection between miR-34b-5p and SRSF1 (Fig. 5B). qRT-PCR and WB results disclosed that SRSF1 mRNA and protein were heightened in gefitinib-sensitive tissue relative to normal tissues, further raised in gefitinib-resistant tissues (Fig. 5C, D). Additionally, Spearman analysis revealed that SRSF1 mRNA was inversely linked to miR-34b-5p in NSCLC tissues (R^2^ = 0.4566; *P* < 0.001) (Fig. 5E). Then, SRSF1 level in NSCLC cells was assessed by qRT-PCR and WB. The data revealed that SRSF1 mRNA and protein levels were clearly enhanced in PC9 and HCC827 contrasted to 16HBE, while SRSF1 mRNA and protein expression were further heightened in HCC827-GR and PC9-GR (Fig. 5F, G). The outcomes manifested that miR-34b-5p restrained NSCLC growth by targeting SRSF1.

### Overexpression of miR-34b-5p alleviates the cell colony formation, apoptosis and gefitinib susceptibility via SRSF1

**Fig. 6:**
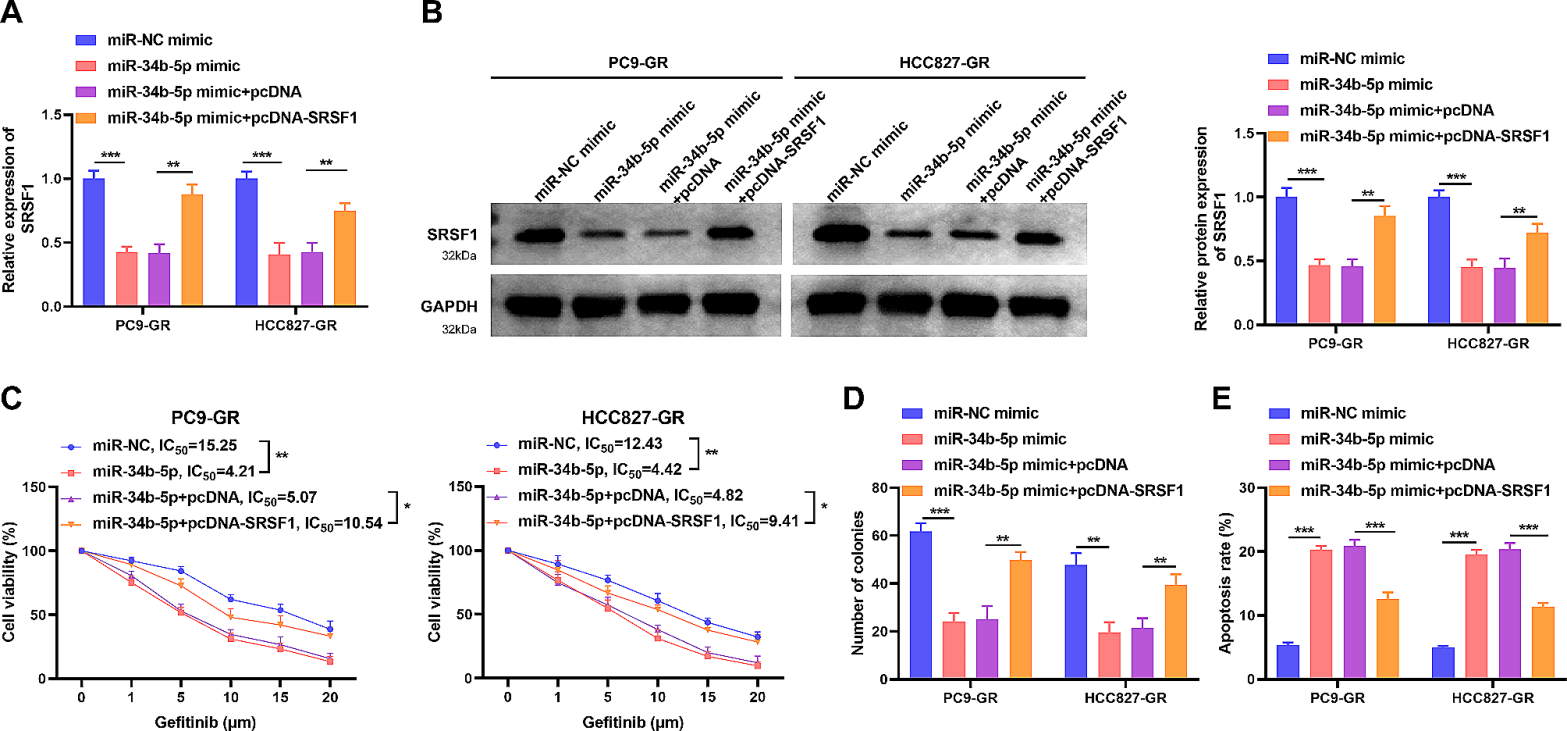
miR-34b-5p overexpression on gefitinib-resistant NSCLC cell colony formation, apoptosis and gefitinib sensitivity was alleviated by SRSF1. PC9-GR and HCC827-GR cells were transfected with mimic NC, miR-34b-5p mimic, miR-34b-5p mimic + pcDNA or miR-34b-5p mimic + pcDNA-SRSF1. (**A-B**) SRSF1 mRNA level and protein level in different treated groups were measured by qRT-PCR (**A**) and western blot (**B**). (**C-E**), cell viability, colony formation ability and apoptosis were measured in different treated groups by CCK-8 assay (**C**), colony formation assay (**D**) and flow cytometry (**E**). *P < 0.05, **P < 0.01, ***P < 0.001

To elucidate whether miR-34b-5p acted by targeting SRSF1 in NSCLC cells, a rescue assay was performed. The transfection efficiency of cells after transfection with pcDNA-SRSF1 was examined by qRT-PCR and WB. miR-34b-5p insertion decreased the SRSF1 mRNA and protein levels, whereas pcDNA-SRSF1 abolished this change, implying that the transfection was prosperous (Fig. 6A-B). Subsequently, the effect of co-transfection of pcDNA-SRSF1 with miR-34b-5p mimic on cell function was examined. Compared to miR-NC, miR-34b-5p mimic evidently suppressed the IC50 value and cell cloning ability of HCC827-GR and PC9-GR, however, SRSF1 introduction reversed this influence of miR-34b-5p mimic on cells (Fig. 6C-D). Furthermore, miR-34b-5p mimic considerably improved the apoptotic capacity, while reversed by insertion of SRSF1 (Fig. 6E). These findings suggested that the impact of miR-34b-5p on cells was owing to SRSF1 introduction.

### circ_0001786 regulates the SRSF1 expression via sponging miR-34b-5p

**Fig. 7:**
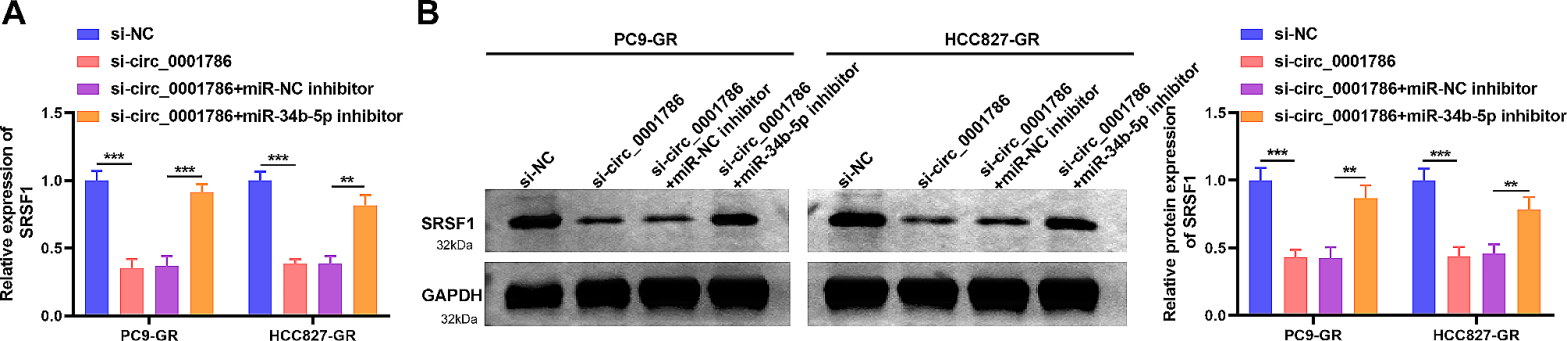
circ_0001786 regulated SRSF1 expression by sponging miR-34b-5p. SRSF1 mRNA and protein levels by qRT-PCR and western blot in PC9-GR and HCC827-GR cells transfected with si-NC, si-circ (si-circ_0001786), si-circ + inhibitor NC or si-circ + miR-34b-5p inhibitor was measured by qRT-PCR (**A**) and western blot (**B**). **P < 0.01, ***P < 0.001

On the basis of the above outcomes, we determined to clarify whether circ_0001786 adjusted the SRSF1 via sponging miR-34b-5p. We transfected si-circ_0001786 together with miR-34b-5p inhibitor into gefitinib-resistant cells and examined SRSF1 expression. We discovered that knockdown of circ_0001786 significantly reduced the SRSF1 mRNA and protein levels in PC9-GR and HCC827-GR, while the impact was inverted by miR-34b-5p inhibitor (Fig. 7A, B). All outcomes confirmed that circ_0001786 adjusted SRSF1 through sponging miR-34b-5p.

## Discussion

Despite the significant advancements in NSCLC diagnosis and treatment over the past few years, its recurrence rate and mortality were still not low [20]. Research suggested that molecularly targeted therapy is a more effective treatment option [21, 22]. Gefitinib, a specific antagonist of receptor tyrosine kinase for epidermal growth factor, had the ability to inhibit tumor angiogenesis, suppress cell division and promote apoptosis [23]. Studies had confirmed its ability to improve clinical symptoms and prolong survival of patients with advanced NSCLC. However, clinical trials had identified that most NSCLC patients with effective gefitinib treatment develop varying degrees of resistance after 6 months of treatment, leading to treatment failure. Resistance to gefitinib had become a critical hinder to lung cancer treatment, so it was necessary to investigate the specific mechanism of its resistance. It had been reported that circRNA was linked to the progression of resistance of NSCLC cells to various drugs, including gefitinib.

Recent developments in the study of circRNAs discovered that a prominent mechanism for circRNAs exerted their functions is to target some tumor-associated miRNAs to play a significant regulatory role [24]. For example, Wu et al. observed that Hsa_circ_0020714 induced immune evasion and resistance to anti-PD-1 immunotherapy in NSCLC by modulating the miR-30a-5p/SOX4 axis [25]. Furthermore, Gao et al. showed that elevated circASCC3 upregulated C5a via spongy miR-432-5p, which facilitated NSCLC progression and immune dysfunction status [26]. In NSCLC, a portion of circRNAs were participated in regulating gefitinib resistance. Such as hsa_circ_0004015 was heightened in NSCLC tissues and regulated growth, invasion, and TKI resistance in NSCLC through the miR-1183/PDPK1 axis [27]. Some scholars revealed that circ_0000567 dissimilarly expressed in gefitinib tolerance NSCLC cells contributed to the growth of gefitinib-resistant cells [28]. However, the effect of circ_0001786 on gefitinib tolerance in NSCLC was unclear. Our experiment revealed that circ_0001786 was dramatically raised in gefitinib-resistant cells and tissues. Functionally, circ_0001786 silencing inhibited cell activity, induced apoptosis and made gefitinib-resistant cells susceptible to gefitinib. The outcomes of this study disclosed that circ_0001786 strengthened the resistance to gefitinib in NSCLC. To better ascertain the mechanism of circ_0001786 on NSCLC gefitinib sensitivity, a target binding relationship between circ_0001786 and miR-34b-5p was identified through the database.

miRNAs could bind to specific sequences in the 3’ UTR of target genes, leading to mRNA reduction or translation suppression. Researches had shown that miRNAs were significantly dissimilarly expressed in NSCLC contrasted with normal lung tissue and were involved in regulating the onset, progression and drug resistance of NSCLC [29]. Lu et al. discovered that miR-15b silencing partly rescued cisplatin tolerance in NSCLC via the GSK-3β/MCL-1 axis [30]. miR-34b-5p was related to kinds of diseases. miR-34b-5p acted a crucial part in the proliferation, migration and chemoresistance of multiple tumor cells by targeting a range of molecules. For instance, miR-34b-5p expression was alleviated and inhibited cell growth and migration by targeting ARHGAP1 in breast cancer [31]. miR-34b-5p also facilitated the tolerance of bladder cancer cell to cisplatin via targeting ABCB1 [32]. The outcomes of this discussion were similar to those of previous studies. miR-34b-5p was diminished in gefitinib-tolerant cells and tissues and inversely associated to the level of circ_0001786. Knockdown of miR-34b-5p abolished the inhibitory of si-circ_0001786 on cell growth and the promotion of apoptosis by si-circ_0001786. To better understand the mechanism, we found SRSF1 to be a downstream target of miR-34b-5p by cross-referencing from four databases (TargetScan, StarBase, miRTarBase, miRTarBase), qRT-PCR, and searching the literature.

The splicing factor SRSF1 was a critical element of the splicing activator SR family, and SRSF1 typically combines with exon splicing promoters to accelerate splicing and to intronic parts to prevent splicing [33]. Apart from its function in splicing, it also played a role in mediating mRNA export and translation. SRSF1 had been discovered as a probable oncogene, highly expressed in a number of malignancies. For example, USP4 and USP15 promoted the growth of lung cancer cells via measuring selective splicing of SRSF1 [34]. SRSF1 inhibited autophagy by adjusting Bcl-x splicing and combining PIK3C3 in lung cancer [35]. circ_000829 acts as an anticancer agent in renal cell carcinoma via inhibiting SRSF1-mediated selective splicing of SLC39A14 [36]. However, the specific mechanism of SRSF1 in NSCLC was not clear. Our research disclosed that SRSF1 was enlarged in gefitinib-tolerant tissues and cells and inversely correlated to miR-34b-5p. In addition, SRSF1 insertion returned the prohibitory influence of miR-34b-5p mimic on cell growth and the promotion of apoptosis by miR-34b-5p mimic. These revealed that circ_0001786 exerted a regulatory effect on gefitinib resistance in NSCLC via targeting miR-34b-5p and thus inducing SRSF1. Nonetheless, the sample size selected in this research was small and less representative. Therefore, we will add the experimental sample size in subsequent experiments to further elucidate the mechanism of circ_0001786 involved in the resistance of NSCLC cells to gefitinib.

In conclusion, our study demonstrated that circ_0001786 induced SRSF1 through targeting miR-34b-5p and had a useful role in facilitating cellular malignant behavior and gefitinib resistance. This study revealed for the first time the role of circ_0001786 in gefitinib-resistant NSCLC, providing new insights into the development of NSCLC in which circ_0001786 becomes resistant to gefitinib chemotherapy.

## Data Availability

The data used to support the findings of this study are available from the corresponding author upon request.

## Abbreviations

NSCLC: Non-small cell lung cancer
EGFR: Epidermal growth factor receptor
EGFR-TKIs: EGFR-tyrosine kinase inhibitors
circRNAs: circular RNAs
miRNA: microRNA
