# Correction: Circ_0001786 facilitates gefitinib resistance and malignant progression in non-small cell lung cancer via miR-34b-5p/SRSF1

**DOI:** 10.1186/s13019-024-02773-0

**Published:** 2024-04-20

**Authors:** Kaobin Ouyang, Dan Xie, Haojie Liao, Ying He, Hailin Xiong

**Affiliations:** Department of Medical Oncology, Huizhou Municipal Central Hospital of Guangdong Province, NO.41 North Eling Road, Huizhou, 516000 Guangdong Province China

**Correction: Journal of Cardiothoracic Surgery (2024) 19:178**

10.1186/s13019-024-02651-9

Following publication of the original article [1], the author’s name ‘Hailin Xiong’ was incorrectly written as ‘Hailing Xiong’.

The original article has been corrected.
